# Supplementary material for: The Effect of Photosensitizer Metalation Incorporated into Arene–Ruthenium Assemblies on Prostate Cancer
Source: Int J Mol Sci. 2023 Sep 2;24(17):13614. doi: 10.3390/ijms241713614 (PMC10488040; doi:10.3390/ijms241713614)
Supplement: Supplementary file 1 [file ijms-24-13614-s001.zip › ijms-2554419-supplementary.pdf]

A

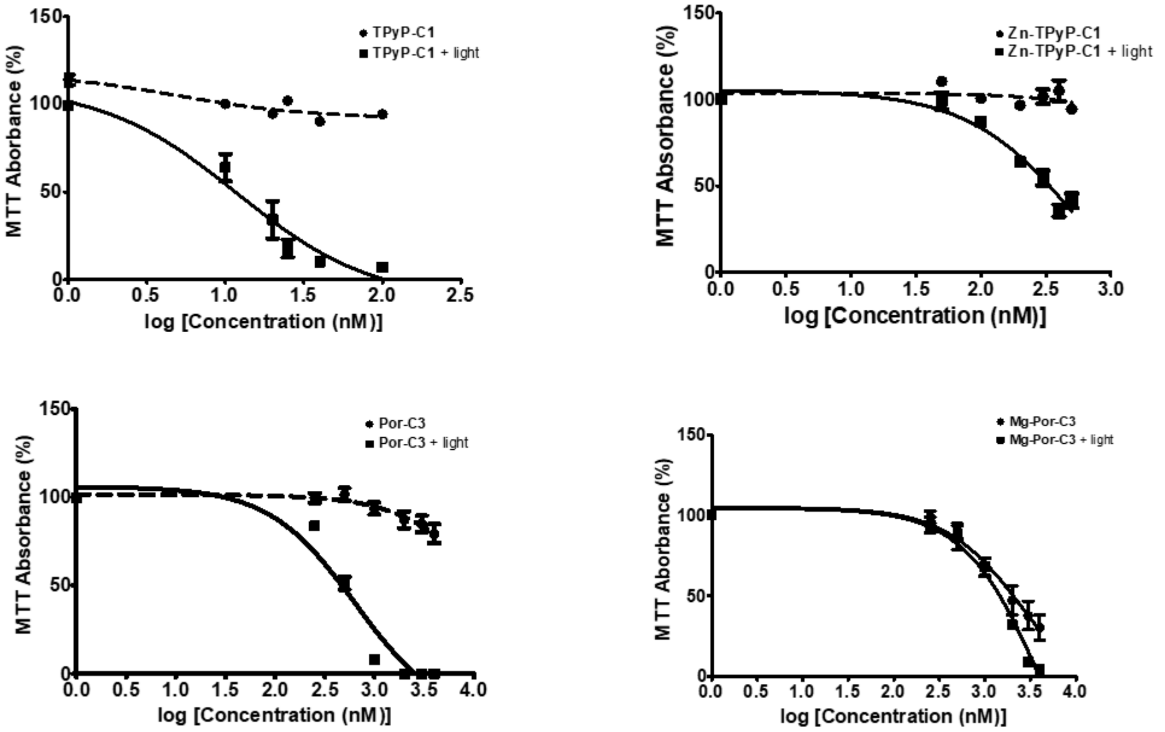
TPyP-C1
TPyP-C1 + light
MTT Absorbance (%)
log [Concentration (nM)]
Zn-TPyP-C1
Zn-TPyP-C1 + light
MTT Absorbance (%)
log [Concentration (nM)]
Por-C3
Por-C3 + light
MTT Absorbance (%)
log [Concentration (nM)]
Mg-Por-C3
Mg-Por-C3 + light
MTT Aborbance (%)
log [Concentration (nM)]

B

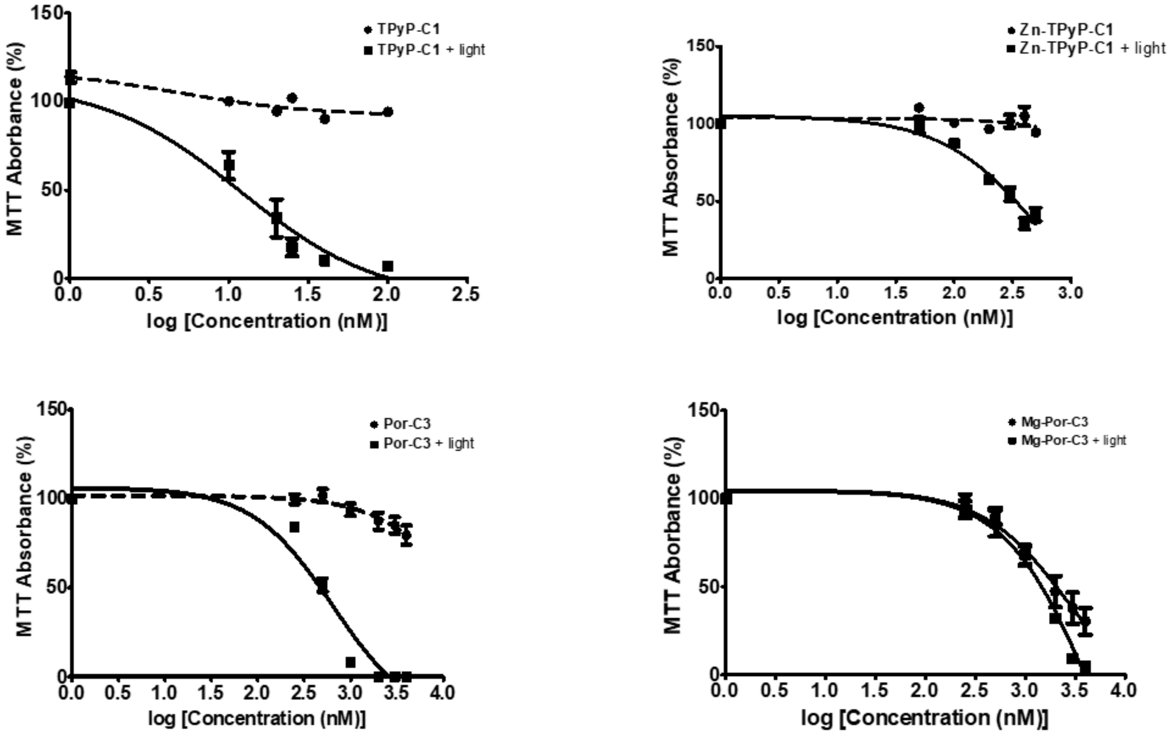
TPyP-C1
TPyP-C1 + light
MTT Absorbance (%)
log [Concentration (nM)]
Zn-TPyP-C1
Zn-TPyP-C1 + light
MTT Absorbance (%)
log [Concentration (nM)]
Por-C3
Por-C3 + light
MTT Absorbance (%)
log [Concentration (nM)]
Mg-Por-C3
Mg-Por-C3 + light
MTT Absorbance (%)
log [Concentration (nM)]

C

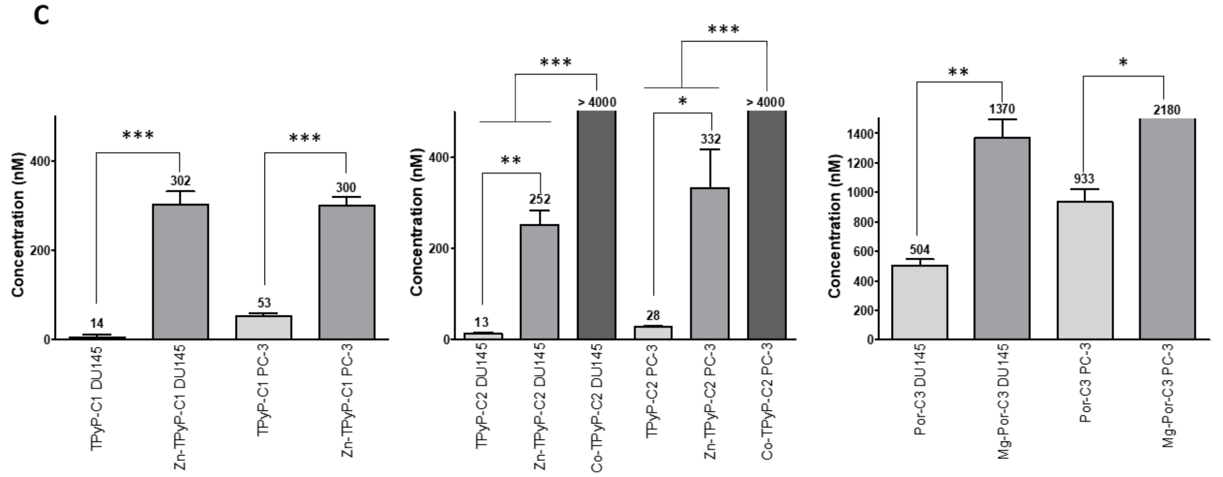

Figure S1. Phototoxicity of PS on prostate cell lines. Cells were cultured in RPMI medium during 24 h. DU 145 were treated or not with compounds **(A) TPyP-C1, Zn-TPyP-C1, Por-C3** or **Mg-Por-C3** and PC-3 **(B) TPyP-C1, Zn-TPyP-C1 Por-C3** or **Mg-Por-C3** for 24 h. Cells were irradiated (630 nm, 75J/cm²) or kept in the dark. Compounds toxicity at 24h was followed by MTT test and IC_{50} values were calculated. Cell viability, was compared to controls. **(C)** Data represent IC_{50} (top of the bar) with irradiation as mean ± SEM (n=3). *p < 0,05; **p < 0,01 and ***p < 0,001.

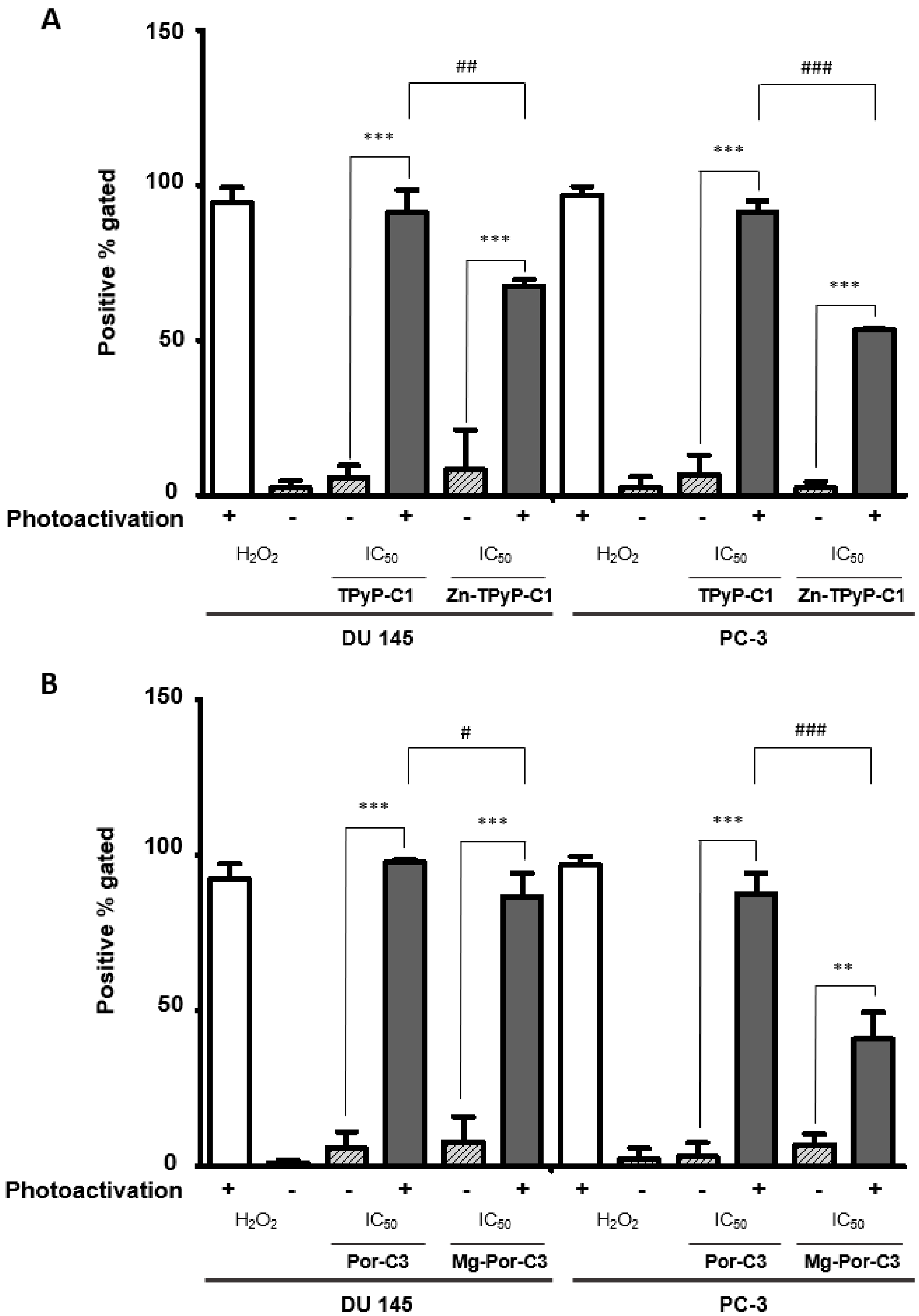

Figure S2. Compounds induced ROS production in human prostate cancer cell lines PC-3 and DU 145. Cells were treated with compounds and photoactivated or not. Intracellular ROS levels using DCFDA staining were measured directly after PDT by flow cytometry. (**A**) Cells were treated with **TPyP-C1 or Zn-TPyP-C1.** (**B**) Cells were treated with **Por-C3 or Mg-Por-C3**. A higher fluorescence intensity resulting from a higher amounts of 2′,7′-dichlorofluorescein (DCF) formation results of a shift to the right. Data are shown as mean ± SEM (n=3). *$p < 0.05$; **$p < 0.01$ and ***$p < 0.001$ relative to compounds or #$p < 0.05$; ##$p < 0.01$; ###$p < 0.001$ relative to other compounds.

A

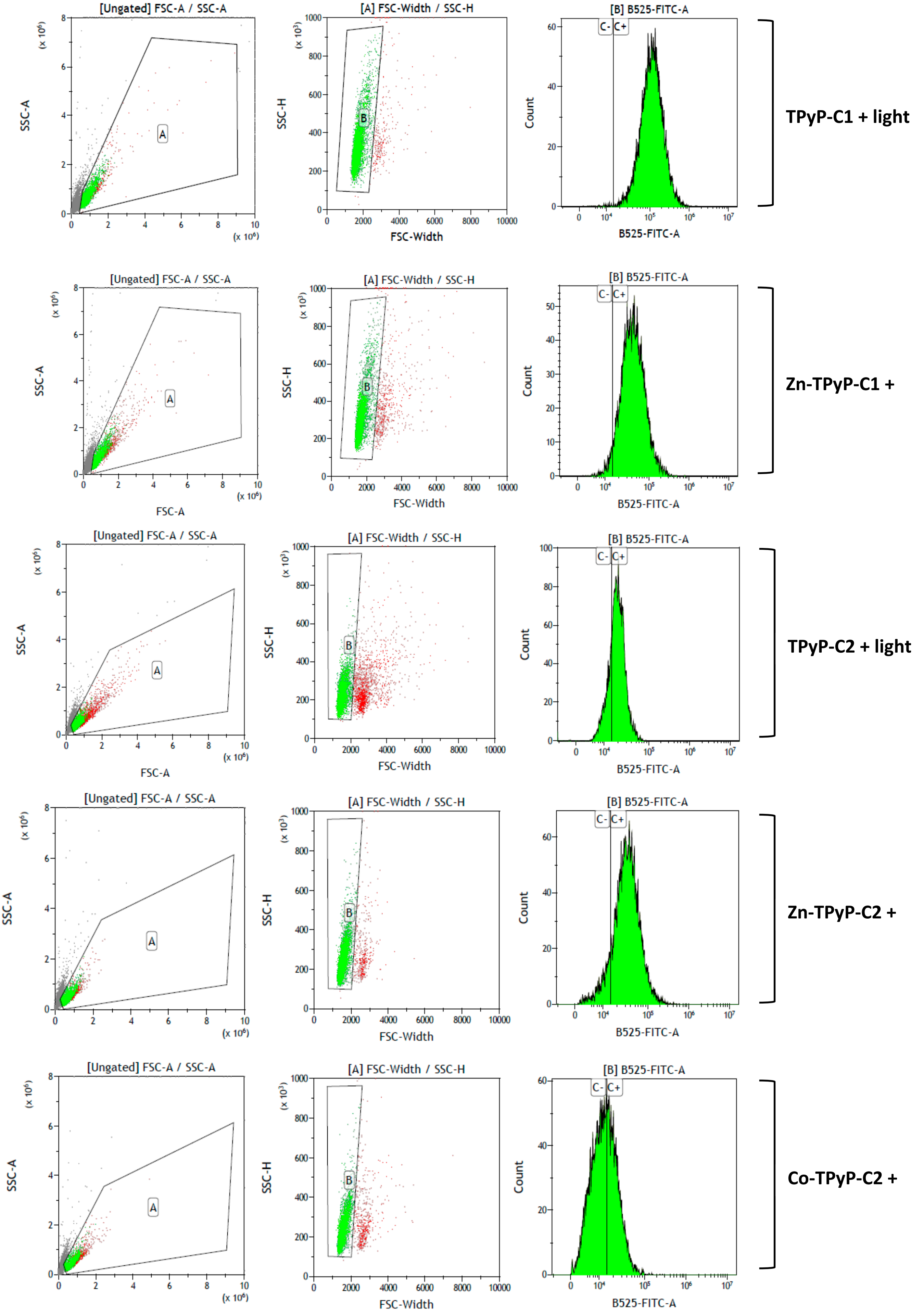
[Ungated] FSC-A / SSC-A
[A] FSC-Width / SSC-H
[B] B525-FITC-A
SSC-A
FSC-A
SSC-H
FSC-Width
Count
B525-FITC-A
C-
C+
TPyP-C1 + light
Zn-TPyP-C1 +
TPyP-C2 + light
Zn-TPyP-C2 +
Co-TPyP-C2 +

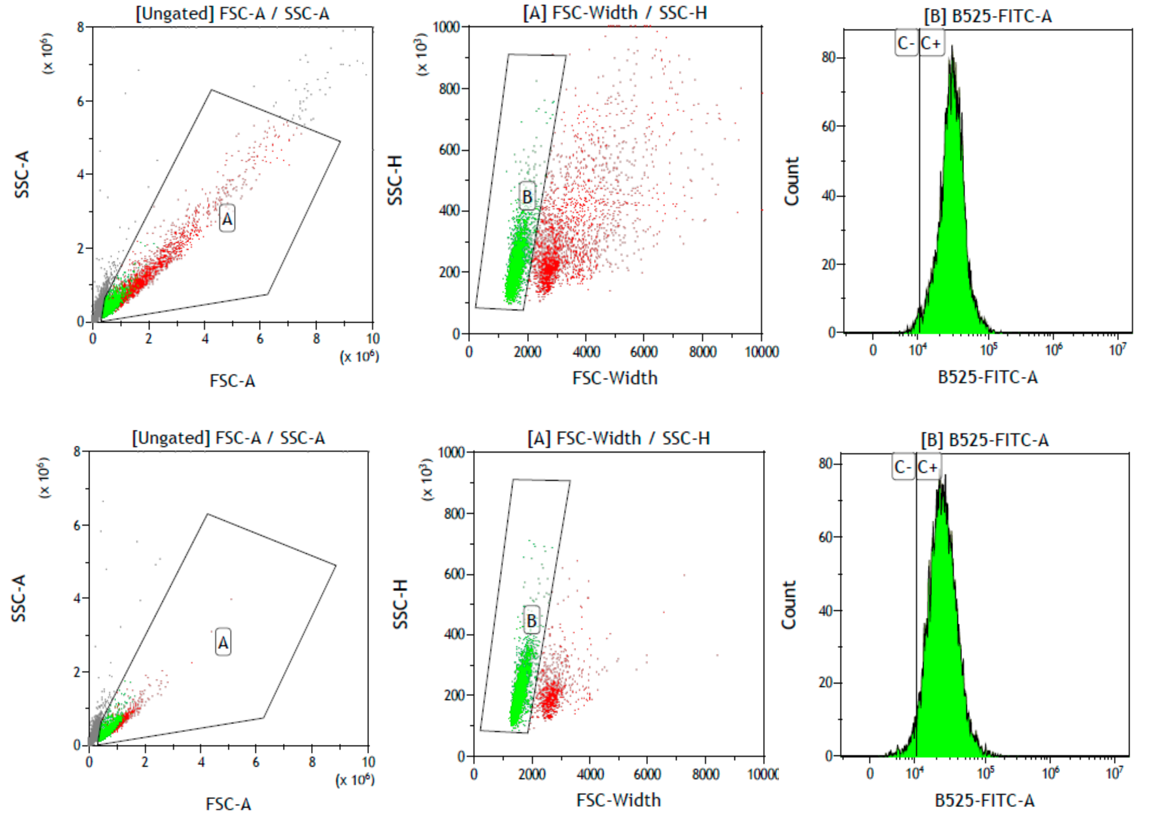

Por-C3 + light

Mg-Por-C3 +

B

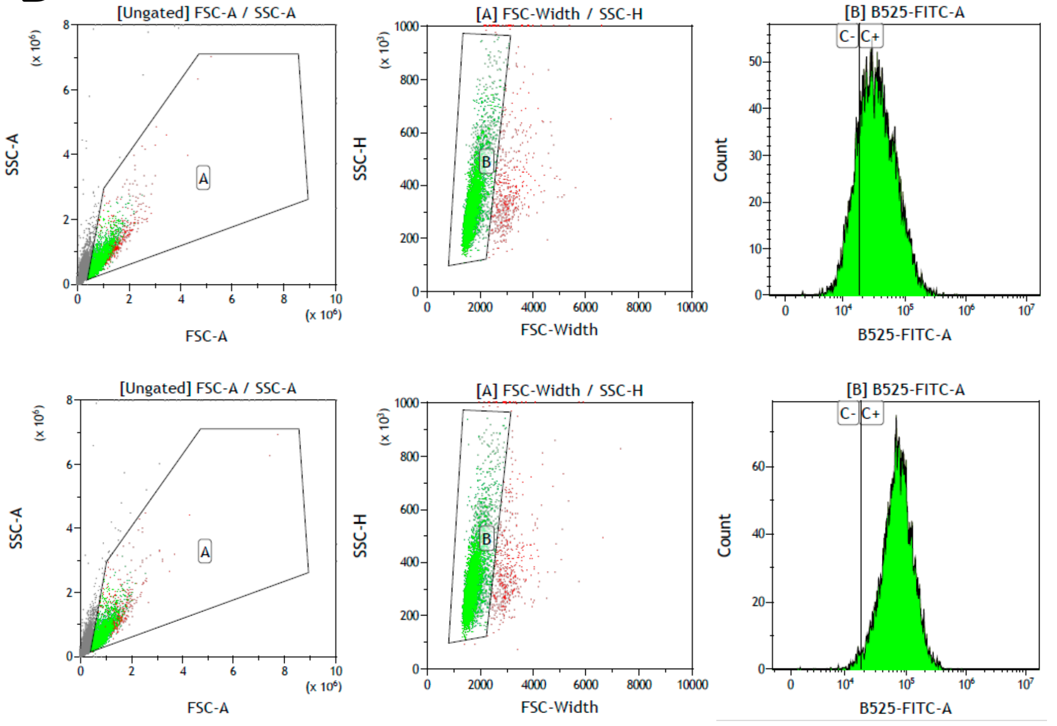

TPyP-C1 + light

Zn-TPyP-C1 +

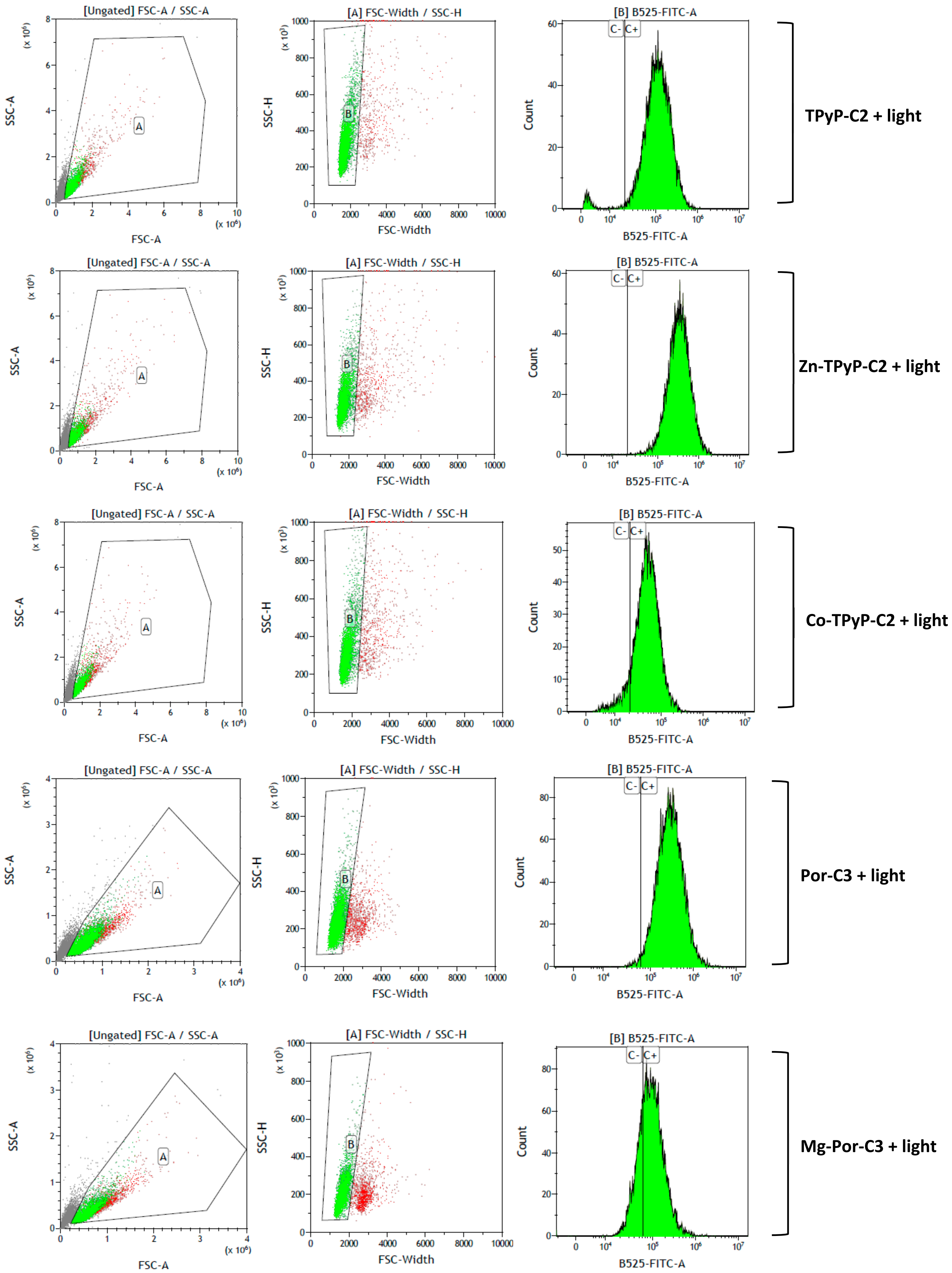
[Ungated] FSC-A / SSC-A
SSC-A
(x 10⁶)
FSC-A
A
[A] FSC-Width / SSC-H
SSC-H
(x 10³)
FSC-Width
B
[B] B525-FITC-A
Count
B525-FITC-A
C-
C+
TPyP-C2 + light
Zn-TPyP-C2 + light
Co-TPyP-C2 + light
Por-C3 + light
Mg-Por-C3 + light

Figure S3. Production of reactive oxygen species (ROS) in DU 145 **(A)** or PC-3 **(B)** cell lines. Histogram overlay ROS production in prostate cancer cells upon irradiation.

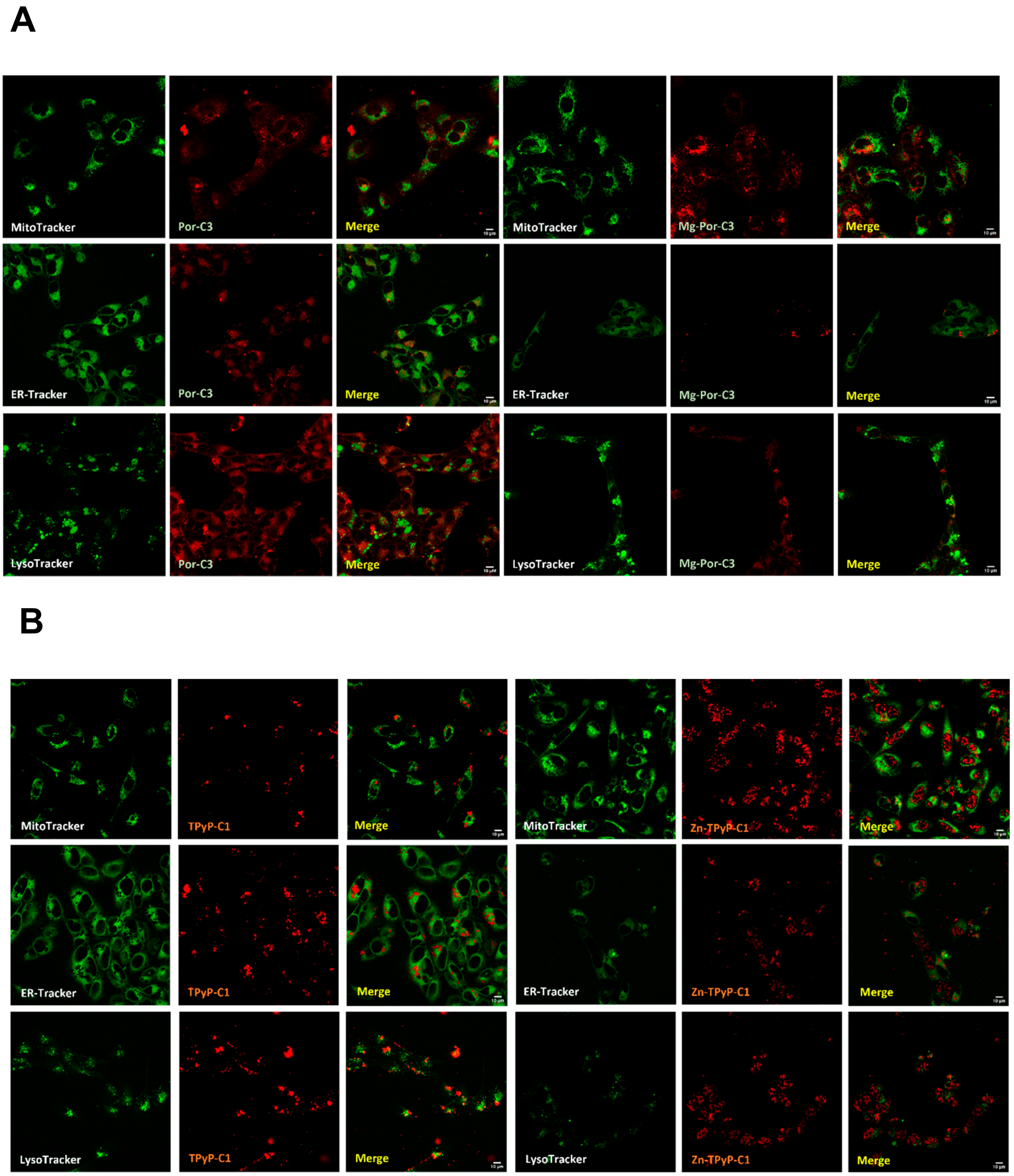
A
MitoTracker
Por-C3
Merge
MitoTracker
Mg-Por-C3
Merge
ER-Tracker
Por-C3
Merge
ER-Tracker
Mg-Por-C3
Merge
LysoTracker
Por-C3
Merge
LysoTracker
Mg-Por-C3
Merge
B
MitoTracker
TPyP-C1
Merge
MitoTracker
Zn-TPyP-C1
Merge
ER-Tracker
TPyP-C1
Merge
ER-Tracker
Zn-TPyP-C1
Merge
LysoTracker
TPyP-C1
Merge
LysoTracker
Zn-TPyP-C1
Merge

C

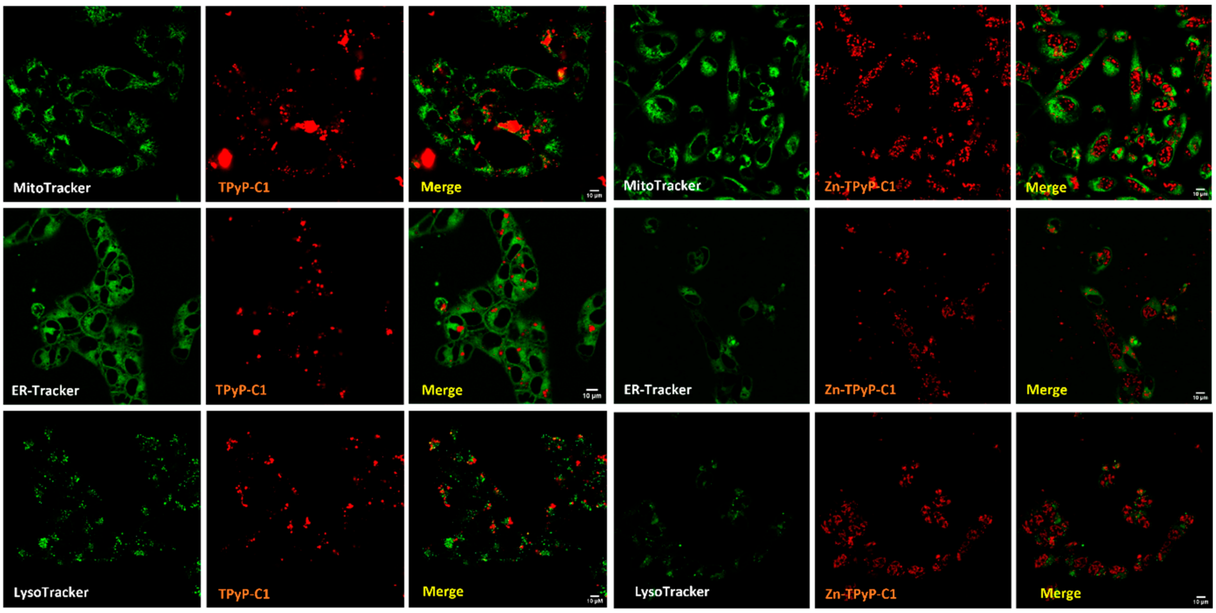

D

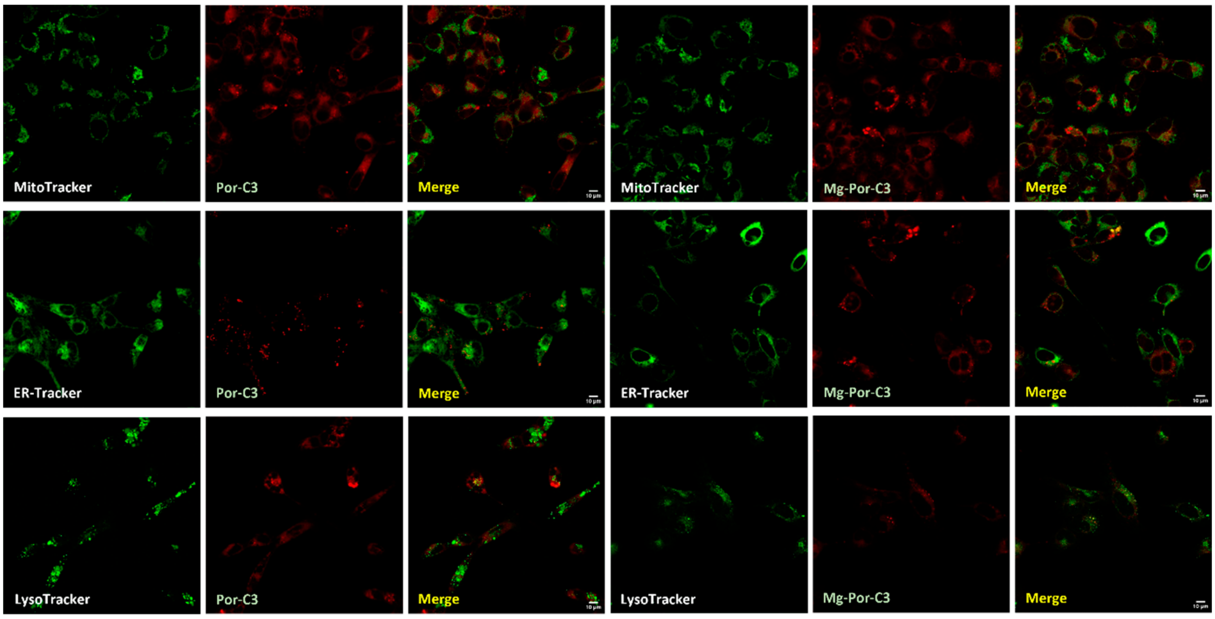

Figure S4. PS localization in human prostate cancer cells. (**A**) DU145 cells treated with **TPyP-C1, or Zn-TPyP-C1**. (**B**) DU 145 cells treated with **Por-C3 or Mg-Por-C3**. (**C**) PC-3 cells treated with **TPyP-C1, or Zn-TPyP-C1**. (**D**) PC-3 cells treated with **Por-C3 or Mg-Por-C3**. PS localization was studied by confocal microscopy and photos were taken with a confocal microscope (laser Zeiss LSM 510 Meta – ×1000). White scale bar represents 10 μM.

A

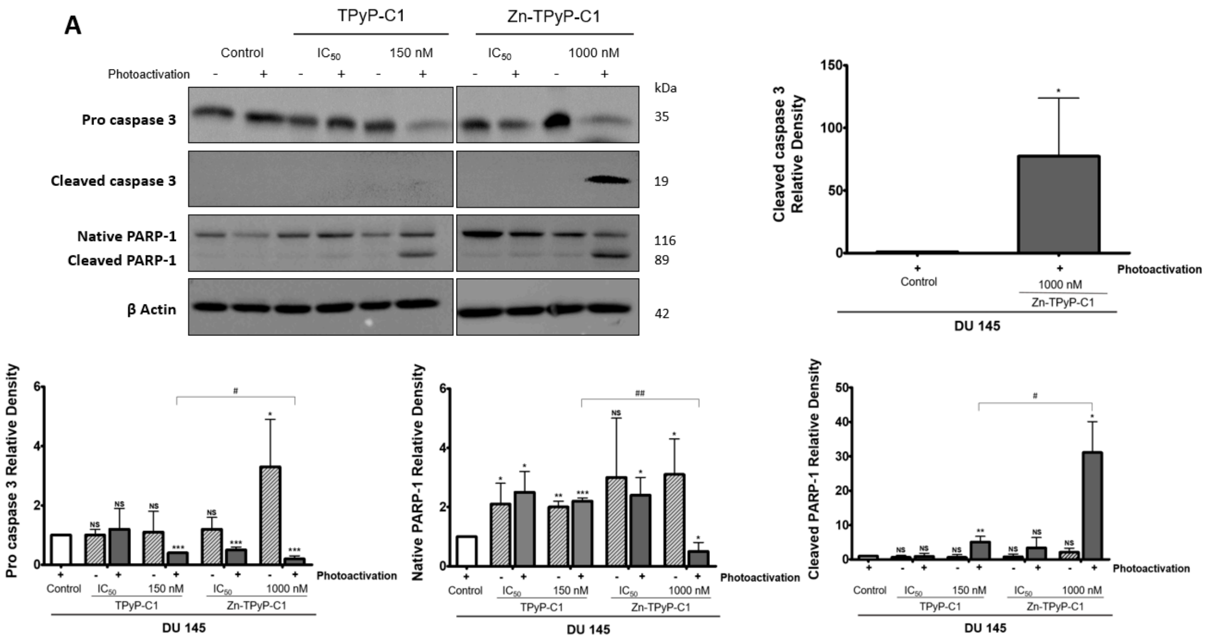

TPyP-C1
Zn-TPyP-C1
Control
IC50
150 nM
1000 nM
Photoactivation
kDa
Pro caspase 3
Cleaved caspase 3
Native PARP-1
Cleaved PARP-1
β Actin
35
19
116
89
42
Cleaved caspase 3 Relative Density
Pro caspase 3 Relative Density
Native PARP-1 Relative Density
Cleaved PARP-1 Relative Density
DU 145

B

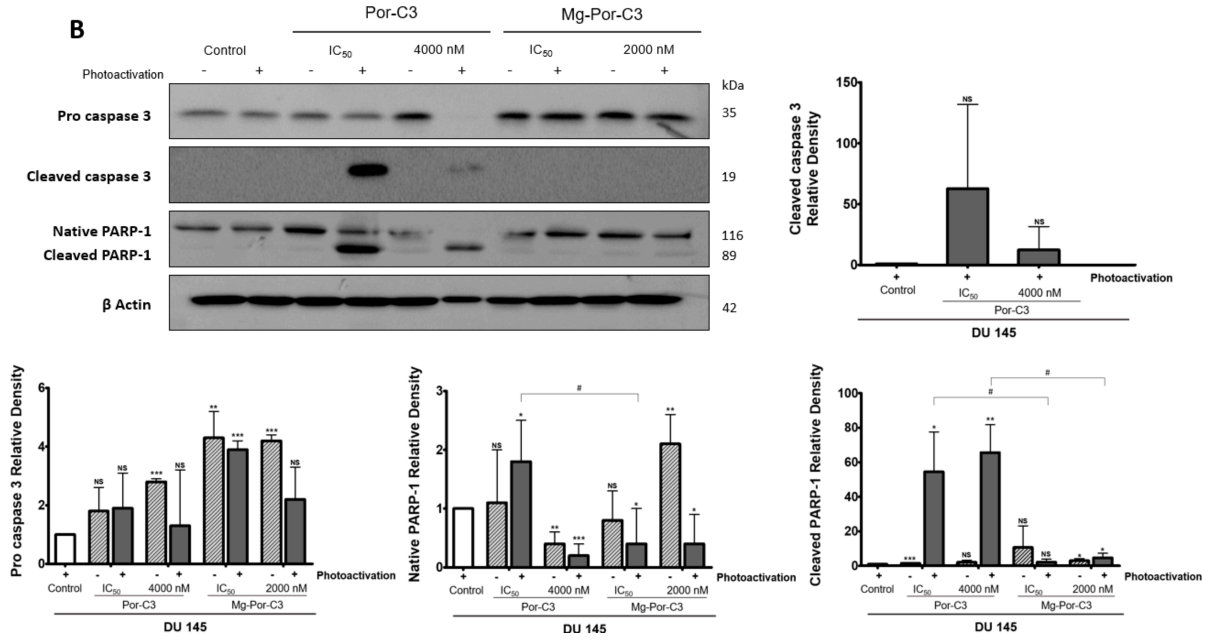

Por-C3
Mg-Por-C3
Control
IC50
4000 nM
2000 nM
Photoactivation
kDa
Pro caspase 3
Cleaved caspase 3
Native PARP-1
Cleaved PARP-1
β Actin
35
19
116
89
42
Cleaved caspase 3 Relative Density
Pro caspase 3 Relative Density
Native PARP-1 Relative Density
Cleaved PARP-1 Relative Density
DU 145

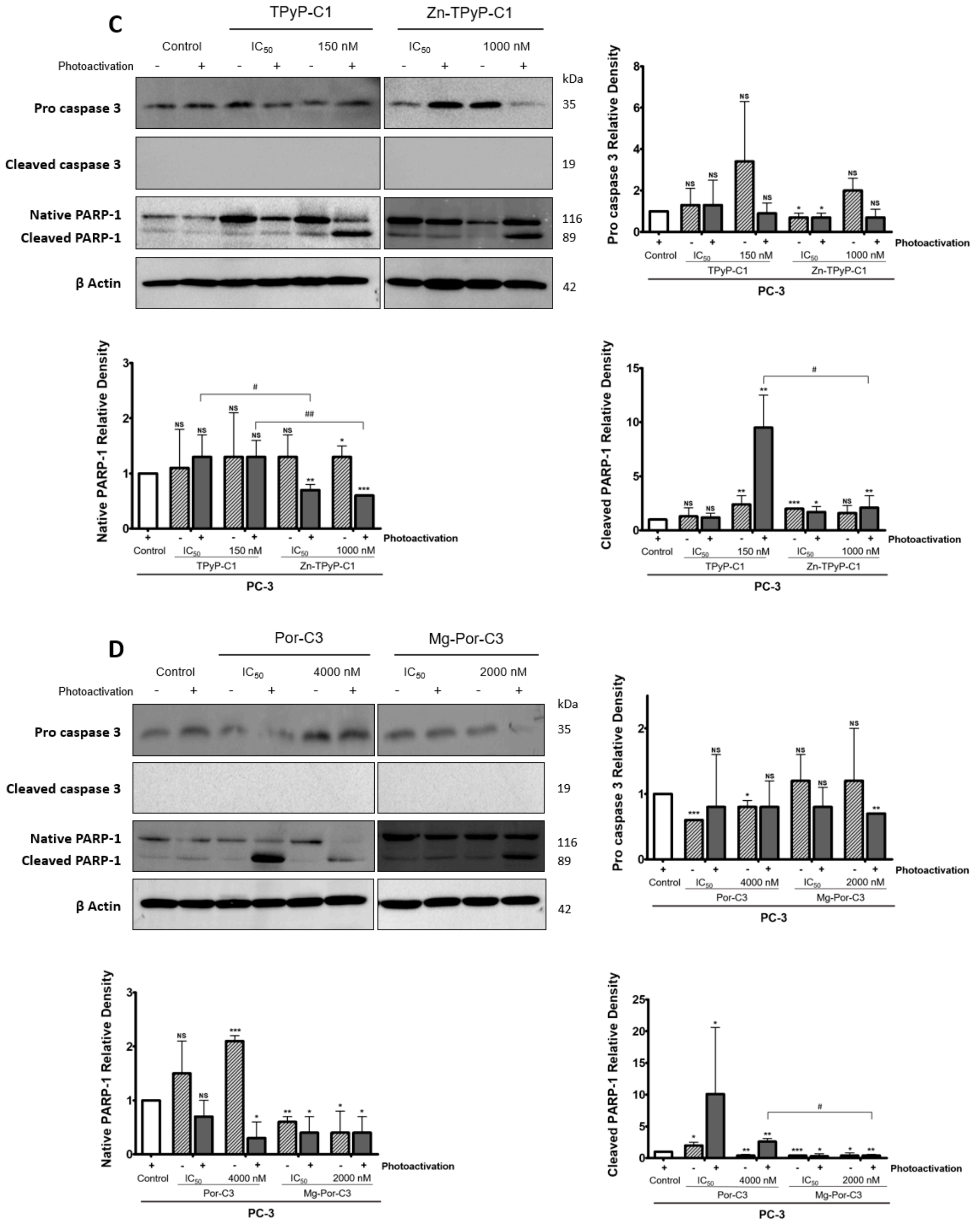

Figure S5. Effects of compounds on human prostate cancer cell lines. **(A)** DU145 cells were treated or not with **TPyP-C1 and Zn-TPyP-C1** **(B)** DU145 cells were treated or not with **Por-C3** and **Mg-Por-C3** **(C)** PC-3 cells were treated or not with **TPyP-C1 and Zn-TPyP-C1** **(D)** PC-3 cells were treated or not with **Por-C3** and **Mg-Por-C3**. Expression of caspase-3 activation and PARP-1 cleavage was analyzed by Western blotting 24 h post-PDT. β-actin was used as a loading control. Quantification of Pro caspase-3; quantification of native PARP-1 and cleavage of PARP-1. **p* < 0.05; ***p* < 0.01 and ****p* < 0.001 relative to control group or #*p* < 0.05; ##*p* < 0.01 relative to compounds.

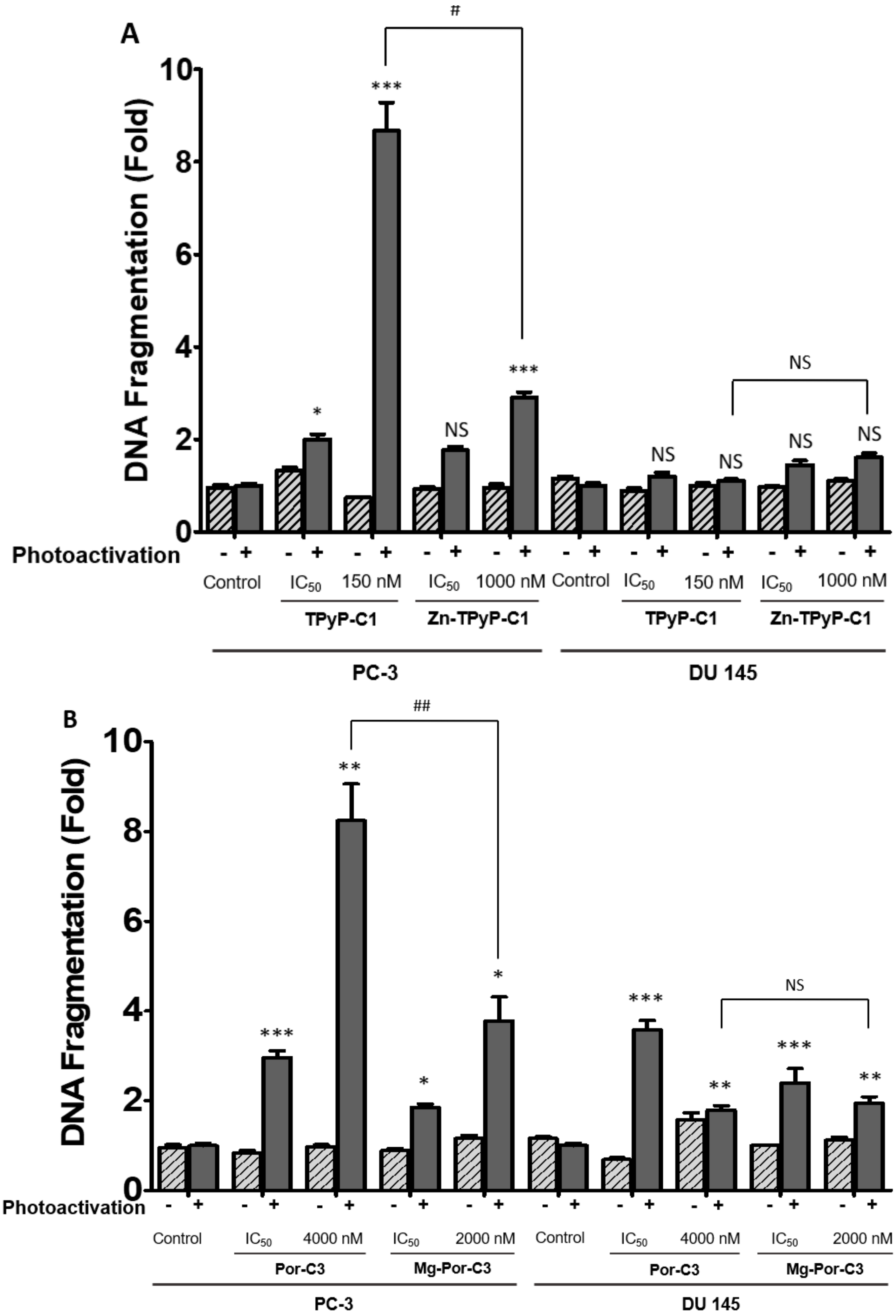

Figure S6. Compounds induced DNA fragmentation through ROS production in prostate cancer cell lines PC-3 and DU 145. DNA fragmentation in cells 24h post PDT was quantified from cytosol extracts by ELISA. Cells were treated with **(A) TPyP-C1, Zn-TPyP-C2** and **Co-TPyP-C2**, **(B) Por-C3** and **Mg-Por-C3**. Results were reported as n-fold compared to light control. Values are expressed as mean ± SEM

(N=3). *$p < 0.05$; **$p < 0.01$ and ***$p < 0.001$ relative to control group or #$p < 0.05$; ##$p < 0.01$ relative to compounds.
